# Efficient Inhibition of Collagen-Induced Platelet Activation and Adhesion by LAIR-2, a Soluble Ig-Like Receptor Family Member

**DOI:** 10.1371/journal.pone.0012174

**Published:** 2010-08-13

**Authors:** Peter J. Lenting, Geertje H. A. Westerlaken, Cécile V. Denis, Jan Willem Akkerman, Linde Meyaard

**Affiliations:** 1 Institut National de la Santé et de la Recherche Médicale (INSERM) U770 & Univ Paris-Sud, Le Kremlin-Bicêtre, France; 2 Department of Clinical Chemistry & Haematology, University Medical Center Utrecht, Utrecht, The Netherlands; 3 Department of Immunology, University Medical Center Utrecht, Utrecht, The Netherlands; Indiana University, United States of America

## Abstract

LAIR-1 (Leukocyte Associated Ig-like Receptor -1) is a collagen receptor that functions as an inhibitory receptor on immune cells. It has a soluble family member, LAIR-2, that also binds collagen and can interfere with LAIR-1/collagen interactions. Collagen is a main initiator for platelet adhesion and aggregation. Here, we explored the potential of soluble LAIR proteins to inhibit thrombus formation *in vitro*. LAIR-2/Fc but not LAIR-1/Fc inhibited collagen-induced platelet aggregation. In addition, LAIR-2/Fc also interfered with platelet adhesion to collagen at low shear rate (300 s^−1^; IC_50_ = 18 µg/ml) and high shear rate (1500 s^−1^; IC_50_ = 30 µg/ml). Additional experiments revealed that LAIR-2/Fc leaves interactions between collagen and α2β1 unaffected, but efficiently prevents binding of collagen to Glycoprotein VI and von Willebrand factor. Thus, LAIR-2/Fc has the capacity to interfere with platelet-collagen interactions mediated by Glycoprotein VI and the VWF/Glycoprotein Ib axis.

## Introduction

The formation of platelet-rich thrombi is a multistep process involving several components [1]. The initial step of thrombus formation requires the capturing of platelets from flowing blood to the exposed subendothelial matrix of an injured vessel wall. Once bound to this matrix, platelets become activated, allowing the formation of platelet-platelet interactions which is needed for thrombus growth. Platelet activation is enhanced via thrombin, a product of the coagulation cascade or via stimulatory agents (such as ADP, epinephrine and thromboxane A2) that are released from platelet storage granules. Finally, this primary activation and aggregation step is followed by a second wave of signals that lead to stabilization of the platelet aggregate [2].

The first step in the recruitment of platelets to the injured vessel wall is mediated by the platelet glycoprotein (Gp) Ib/IX/V receptor complex, one of the most abundant receptors present at the platelet surface. The main ligand for this GpIb/IX/V complex is von Willebrand factor (VWF), a multimeric plasma protein that functions as a molecular bridge between components of the exposed subendothelial matrix (in particular collagen) and the platelet GpIb/IX/V complex. The interaction between VWF and GpIb/IX/V is of importance not only within arterial and/or stenosed vessels, but also within venous vessels. Indeed, studies using VWF-deficient mice have shown that the absence of VWF is associated with delayed platelet adhesion and defective thrombus formation in vessels with high and low shear rates [3], [4].

Interactions between platelets and subendothelial collagen structures also occur in a direct manner. Two main collagen receptors have been identified on platelets: the integrin α2β1 and a member of the immunoglobulin superfamily, glycoprotein VI (GpVI) [5]. Defining the respective roles of both receptors in the platelet-collagen interaction is still a matter of debate [6]. At lower shear rates, both receptors have the potential to contribute to the adhesion of platelets to collagen. Under conditions of high blood shear (>1500 s^−1^), the contribution of both receptors to initial platelet adhesion is negligible and adhesion is fully dependent on VWF. This does not mean, however, that both receptors are of minor importance. Both GpVI and α2β1 have been shown to be required for stable adhesion of platelets to a collagen surface. In addition, both receptors are coupled to intracellular signaling pathways that contribute to further platelet activation and aggregation as well as the stabilization of platelet aggregates [6].

We identified the inhibitory immune receptor LAIR-1 as a high affinity receptor for collagen [7], [8]. In addition, its soluble family member LAIR-2 is capable of binding collagen and is a potent antagonist of the LAIR-1/collagen interaction [9]. Since multiple binding sites for LAIR-1 and LAIR-2 are present on both human collagens I and III [10], we hypothesized that soluble LAIR proteins are capable of interfering with collagen-platelet interactions. Surprisingly, we observed that LAIR-2 but not LAIR-1 efficiently interferes with collagen-dependent platelet aggregation and adhesion.

## Materials and Methods

### Ethics statement

For donation of peripheral blood, all donors gave written informed consent and approval was obtained from the Institutional Review Board at University Medical Center Utrecht.

### Cells and reagents

Blood was collected from healthy volunteers, who did not use aspirin in the preceding 10 days, in 1/10 volume of 0.13 M sodium citrate and used for the preparation of platelet-rich plasma (PRP). For FACS staining, PRP was supplemented with 0.1 vol ACD (2.5% tri-sodium citrate, 1.5% citric acid and 2.0% D-glucose). After washing with Hepes-Tyrode buffer (0.15 mM NaCl, 5 mM KCl, 0.5 mM Na_2_HPO_4_, 1 mM MgSO_4_, 10 mM Hepes, pH 6.5) supplemented with prostaglandin I_2_ (10 ng/ml final concentration), platelets were resuspended in Hepes-Tyrode buffer (pH 7.4). CHO-cells expressing human α2β1 were a kind gift from Dr. H. Deckmyn, University of Leuven, Belgium [11]. K562-cells transfected with LAIR-1 were described previously [7]. GpVI-expressing Jurkat-cells were prepared a follows: GpVI cDNA (a kind gift of Dr M. Tomlinson, University of Birmingham) was cloned into pMX retroviral vector. Retroviral-based constructs were packaged using the pCL-ampho system [7] and the virus was used to infect Jurkat-cells. Three days after transduction, transfectants expressing GPVI were sorted for high expression on the cell surface using a flow cytometer (FACSAria; BD Biosciences). For FACS analysis, antibodies recognizing the α2-subunit of human α2β1 (CD49b, Clone AK-7, Biolegend), human GpVI (a kind gift from Dr M. Kahn, University of Pennsylvania), human LAIR-1 (Clone DX26, BD Biosciences) and CD62L (BD Biosciences) were used. For Western blotting the following antibodies were used: in-house mouse monoclonal antibody against human LAIR-1 (clone 8A8), mouse monoclonal antibody against human LAIR-2 (clone 3H12, kindly provided by Dr. B. Jin, Fourth Military Medical University, Xi’an, China), and polyclonal rabbit anti human GpVI (kindly provided by Dr. P.A. Smethurst, University of Cambridge, UK). For inhibition studies using Fc-fusion proteins, recombinant chimeric proteins of the extracellular domain of hLAIR-1 or LAIR-2 fused to the Fc region of human IgG1 (LAIR-1/Fc and LAIR-2/Fc, respectively) were prepared as described previously [12]. A chimeric construct of the Signal Inhibitory Receptor on Leukocytes-1 (SIRL-1) fused to the Fc region of human IgG1 (SIRL-1/Fc) was used as control Fc protein. SIRL-1 is an Ig-like receptor with one extracellular Ig-domain and displays structural homology to LAIR-1 and LAIR-2 [13].

### Platelet adherence under flow

Human placenta type III collagen (Sigma) was immobilized onto Thermanox® coverslips by a coating procedure resulting in a concentration of 10 µg/cm^2^. Coverslips were subsequently blocked (16–20 h at 4°C) with 1% human albumin in phosphate-buffered saline (PBS). Perfusions were carried out using citrated human whole blood in a triplicate single-passage parallel-plate perfusion chamber as described [14] in the absence or presence of LAIR-1/Fc, LAIR-2/Fc or control SIRL-1/Fc. Blood was perfused over collagen-coated coverslips for 5 min at various shear rates. After perfusion, slides were washed with Hepes buffer (10 mM Hepes, 150 mM NaCl, pH 7.4) and fixed in 0.5% glutaraldehyde in PBS. Slides were dehydrated in methanol and stained with May-Grünwald and Giemsa. Platelet adhesion was evaluated using computer-assisted analysis with OPTIMAS 6.0 software (Dutch Vision Systems (DVS), Breda, The Netherlands). Platelet attachment to surface-bound collagen was expressed as percentage surface coverage.

### Platelet aggregation

Aggregation studies were carried out with PRP (2–2.5×10^5^ platelets/ml) using an optical aggregometer (Chronolog, Havertown PA, USA). Platelet poor plasma was used to set baseline. Various concentrations of Horm collagen (Nycomed) were pre-incubated with indicated amounts of Fc-fusion proteins for 10 min at 37°C and incubated with PRP for 15 min at 37°C. As control for collagen-independent aggregation, 50 µM thrombin receptor activating peptide (TRAP) was added to PRP.

### Collagen binding to LAIR-1, GpVI and α2β1

Collagen binding to transfectants was assessed by flow cytometry as described previously [11]. In brief, collagen I was labeled with FITC (Molecular Probes) in 0.05 M H_3_BO_3_, 0.2 M NaCl (pH 9.2). After two hours, collagen was dialysed overnight against 0.1 M Tris-HCl, 0.2 M NaCl (pH 7.4) to remove unbound FITC. K562-cells stably transduced with LAIR-1 and Jurkat-cells stably transduced with GPVI were incubated with FITC-labeled collagen for 20 min. To activate α2β1 receptor, CHO-cells expressing α2β1 were incubated in the presence of 1 mM MnCl_2_. After 1 h at 37°C, cells were washed with PBS/10 mM NHCl_4_ and stained with FITC-labeled collagen. Cells were analyzed by flow cytometry (LSRII, BD Biosciences).

### VWF binding to collagen

Microtiter wells were coated with collagen I or collagen III (50 µg/ml) in PBS (pH 7.4) for 16 h at 4°C. After washing three times with PBS/0.01% Tween-20, wells were incubated with purified plasma-derived VWF (0.1 µg/ml) in the absence or presence of recombinant LAIR-1/Fc, LAIR-2/Fc or SIRL-1/Fc (0-100 µg/ml for 1.5 h at 37°C. After washing three times with PBS/0.01% Tween-20, bound VWF was determined via incubation with horseradish-conjugated polyclonal anti-human VWF antibodies (DAKO, Denmark), 1∶3000 diluted in PBS/0.01% Tween-20 for 1.5 h at 37°C. After three washes with PBS/0.01% Tween-20, bound antibodies detecting residual VWF were visualized via incubation with 3-3′-5-5′-tetramethylbenzidine.

## Results

### Platelets do not express LAIR-1 or its soluble counterpart LAIR-2

**Figure 1:**
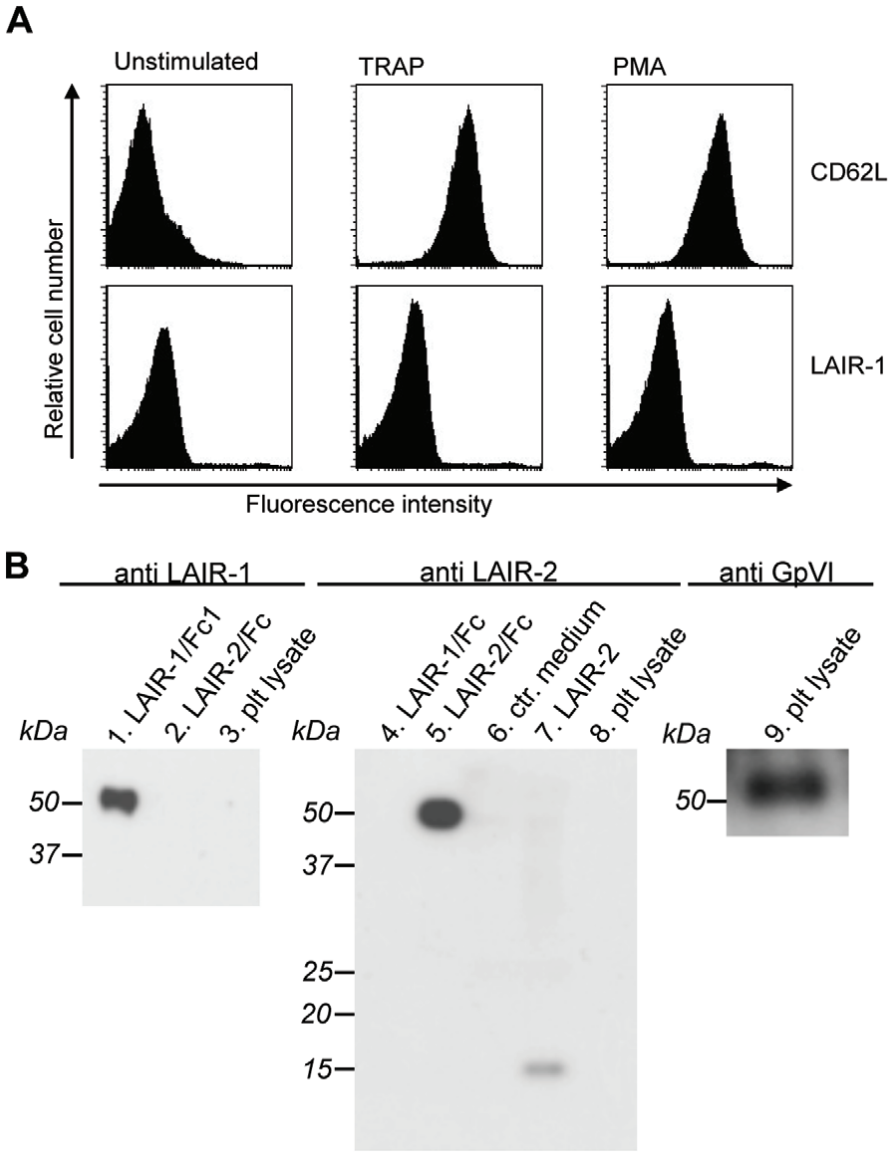
Platelets do not express LAIR-1 or LAIR-2. *Panel* A: Flowcytometric analysis of washed platelets (2×10E5/µl), unstimulated or stimulated for 5 min at RT with 10 µM TRAP or 5000 nM PMA. Upper panels represent staining for CD62L, lower panels represent LAIR-1 staining. *Panel* B: Western blots containing whole platelet lysates and controls were incubated with antibodies against LAIR-1 (*lanes 1–3*), LAIR-2 (*lanes 4–8*) or GpVI (*lane 9*). *Lane 1:* purified LAIR-1/Fc; *lane 2:* purified LAIR-2/Fc; *lane 3:* whole platelet lysate; *lane 4:* purified LAIR-1/Fc; *lane 5:* purified LAIR-2/Fc; *lane 6:* conditioned medium of non-transfected human 293T cells; *lane 7:* conditioned medium of human 293T cells secreting LAIR-2; *lane 8:* whole platelet lysate; *lane 9:* whole platelet lysate.

LAIR-1 and LAIR-2 are both collagen-binding proteins, which are expressed by most haematopoietic cells [15]. Since binding of platelets to the collagen-matrix is an important event in the haemostatic process, we tested if platelets express LAIR-1 and/or LAIR-2. Since we previously observed that unstimulated platelets do not have surface expression of LAIR-1 ([16] and Steevels et al, Manuscript submitted for publication), we now tested whether upon activation platelets LAIR-1 expression was induced. Whereas platelets stimulated with TRAP stained positive for CD62L when analyzed by flow cytometry, no surface LAIR-1 was detected (Fig. 1A). LAIR-1 surface expression was also undetectable on platelets stimulated by collagen or ADP (data not shown). The presence of LAIR-1 or LAIR-2 in platelets was also assessed using Western blotting of whole platelet lysates. Whereas the presence of GpVI in lysates was readily detected, no LAIR-1 or LAIR-2 protein could be detected (Fig. 1B). This let us to conclude that platelets contain no detectable LAIR protein.

### LAIR-2/Fc but not LAIR-1/Fc inhibits platelet aggregation

**Figure 2:**
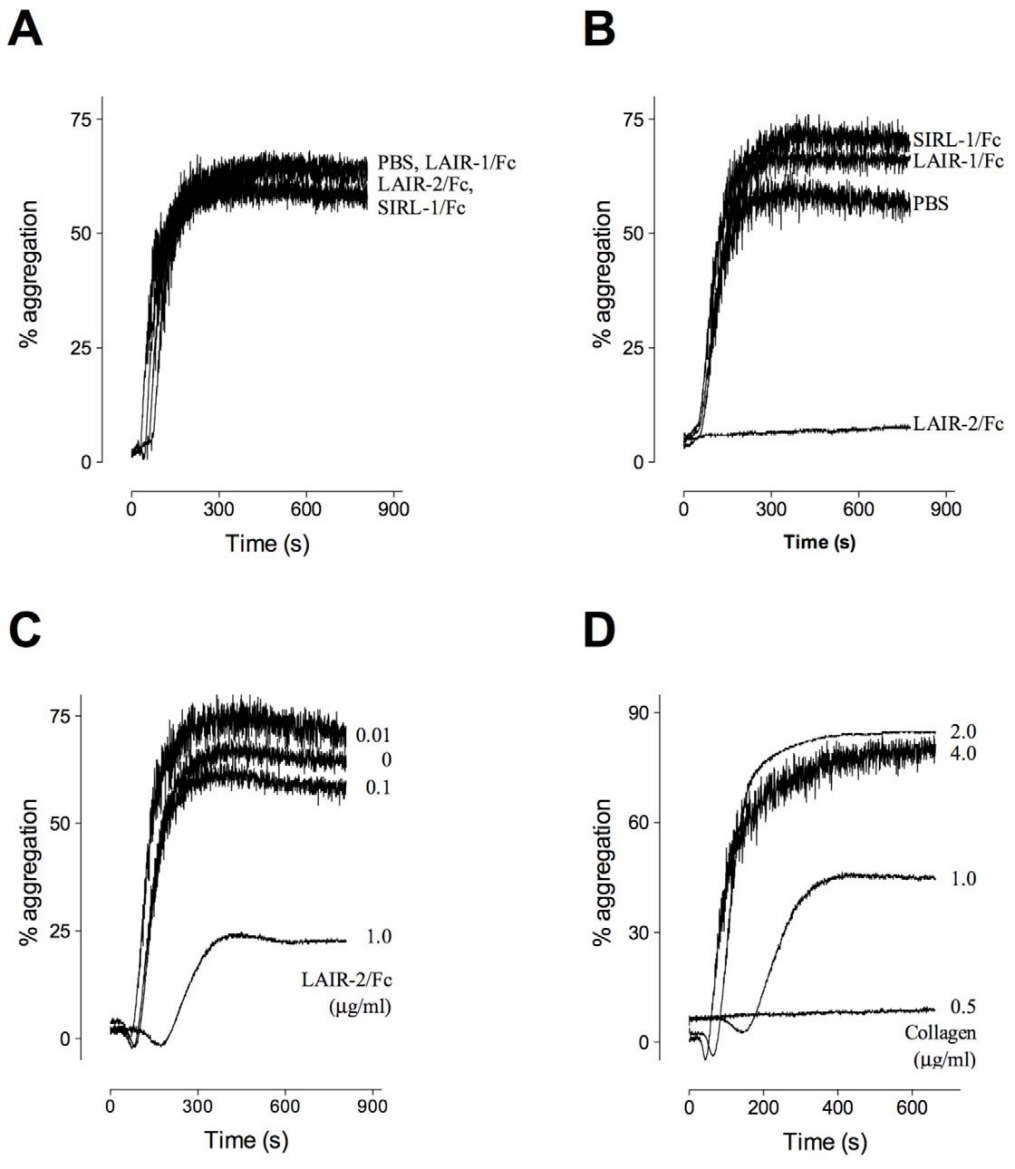
LAIR-2/Fc inhibits collagen but not TRAP-induced platelet aggregation. Aggregation of platelet rich plasma (PRP) in response to collagen was measured using an optical aggregometer. *Panel* A: Platelet aggregation in response to 50 µM TRAP alone (PBS) or in the presence of 100 µg/ml LAIR-1/Fc, LAIR-2/Fc or SIRL-1/Fc. *Panel* B: Platelet aggregation in response to collagen (1 µg/ml) alone (PBS) or in the presence of 100 µg/ml LAIR-1/F, LAIR-2/Fc or SIRL-1/Fc. *Panel* C: Platelet aggregation in response to collagen (1 µg/ml) alone (PBS) or in the presence of 0.01 µg/ml, 0.1 µg/ml or 1.0 µg/ml LAIR-2/Fc. *Panel* D: Platelet aggregation in response to 0.5 µg/ml, 1 µg/ml, 2 µg/ml or 4 µg/ml collagen in the presence of 1.0 µg/ml LAIR-2/Fc.

LAIR-molecules share binding sites on collagens with GpVI [7], [10], [8]. We therefore considered the possibility that soluble LAIR-variants (LAIR-2 and/or recombinant derivatives of LAIR-1) may be used to interfere with platelet-collagen interactions. This was first tested in collagen- and TRAP-induced platelet aggregation experiments. PRP was incubated in the absence or presence of LAIR-1/Fc, LAIR-2/Fc or control protein SIRL-1/Fc (100 µg/ml). None of the tested proteins affected TRAP-induced platelet aggregation (Fig. 2A). Collagen-induced aggregation was also unaffected in the presence of LAIR-1/Fc or control protein SIRL-1/Fc (Fig. 2B). In contrast, the presence of LAIR-2/Fc resulted in complete inhibition of platelet aggregation (Fig. 2B). We next determined the minimal inhibiting dose of LAIR-2/Fc. Platelet aggregation in response to collagen (1.0 µg/ml) was performed in the absence or presence of various concentrations of LAIR-2/Fc (0.01, 0.1 and 1.0 µg/ml). Whereas platelet aggregation was unaffected in the presence of 0.01 µg/ml and 0.1 µg/ml LAIR-2/Fc, a marked reduction in platelet aggregation of more than 50% was observed in the presence of 1.0 µg/ml LAIR-2/Fc (Fig. 2C). In addition, a concentration of 1.0 µg/ml LAIR-2/Fc was able to interfere with platelet aggregation in response to 0.5 µg/ml and 1.0 µg/ml collagen, but not in the presence of 2.0 µg/ml and 4 µg/ml (Fig. 2D). Taken together, these data indicate that LAIR-2/Fc but not LAIR-1/Fc is able to interfere with collagen-induced platelet aggregation.

### LAIR-2/Fc but not LAIR-1/Fc inhibits platelet adhesion to collagen

**Figure 3:**
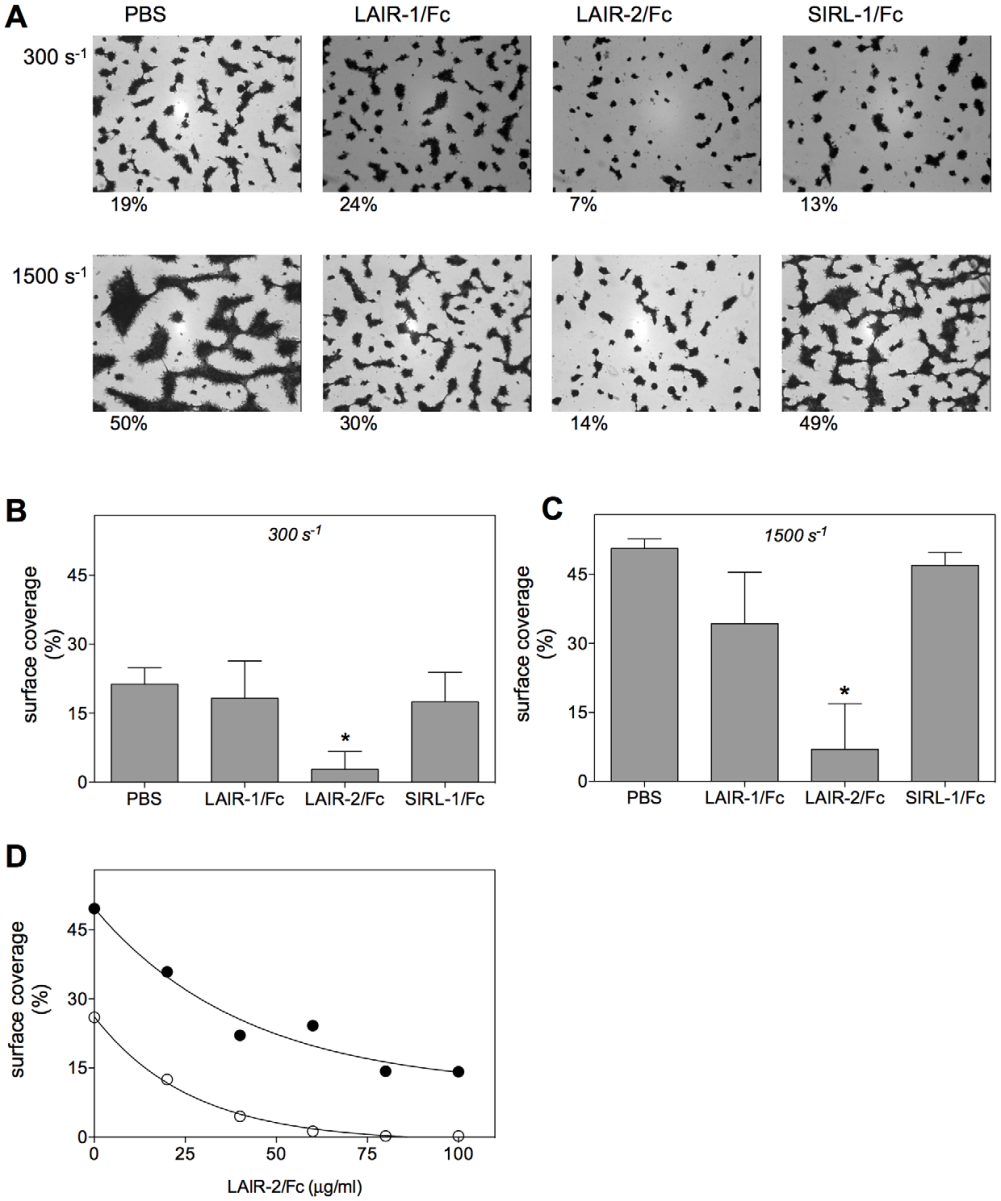
LAIR-2/Fc inhibits adhesion of platelets to collagen under flow conditions. *Panel* A: Collagen type III-coated coverslips were perfused with whole blood in the absence or presence of 100 µg/ml soluble LAIR-1/Fc, LAIR-2/Fc or SIRL-1/Fc. Representative pictures are shown. Perfusion was performed at a shear rate of 300 s^−1^ (upper panels) or 1500 s^−1^ (lower panels). Percentages below figures indicate percentage surface-coverage for each individual photo. *Panels* B & C: Quantitative representation of surface coverage of collagen type III-coated coverslips that were perfused in the absence or presence of 100 µg/ml soluble LAIR-1/Fc, LAIR-2/Fc or SIRL-1/Fc at 300 s^−1^ (*panel* B) or 1500 s^−1^ (*panel* C). Data represent mean±SD of three independent perfusions. *Panel* D: Dose dependent inhibition of surface platelet coverage in the presence of LAIR-2/Fc at shear rates of 300 s^−1^ (open symbols) or 1500 s^−1^ (closed symbols). *: *p*<0.005.

In another series of experiments, we tested the potential of LAIR-1/Fc and LAIR-2/Fc to interfere with the adhesion of platelets to collagen surfaces under conditions of flow. Glass coverslips coated with collagen type III were perfused with citrated whole blood in the absence or presence of LAIR-1/Fc, LAIR-2/Fc or control protein SIRL-1/Fc (100 µg/ml; Fig. 3A). Quantitative analysis revealed that a surface coverage of 21.3±3.6% (mean±SD) was obtained in the absence of these proteins when blood was perfused at low shear rate (300 s^−1^; Fig. 3B). A similar surface coverage was found when LAIR-1/Fc or SIRL-1/Fc were added (18.2±8.1% and 17.5±6.4%, respectively). In contrast, surface coverage was reduced to 2.8±3.8% (p<0.005) in the presence of LAIR-2/Fc (Fig. 3B). At high shear rate (1500 s^−1^), surface coverage increased to 50.7±2.1% when performed in the absence of the Fc-fusion proteins (Fig. 3C). Again, addition of control protein SIRL-1/Fc resulted in a similar coverage (47.0±2.8%). Surface coverage was slightly but not significantly reduced in the presence of LAIR-1/Fc (34.3±11.2%; p = 0.067), and strongly reduced to 7.0±9.9% (p<0.005) in the presence of LAIR-2/Fc (Fig. 3C). The inhibitory potential of LAIR-2/Fc was then assessed in more detail in additional flow adhesion experiments. As depicted in Fig. 3D, half-maximal inhibition was obtained at 18 µg/ml and 30 µg/ml LAIR-2/Fc at 300 s^−1^ and 1500 s^−1^, respectively. Apparently, LAIR-2/Fc not only efficiently interferes with collagen-induced platelet aggregation, but also inhibits platelet-collagen interactions at both low and high shear rates.

### LAIR-2 interferes with GpVI and VWF but not α2β1 binding to collagen

**Figure 4:**
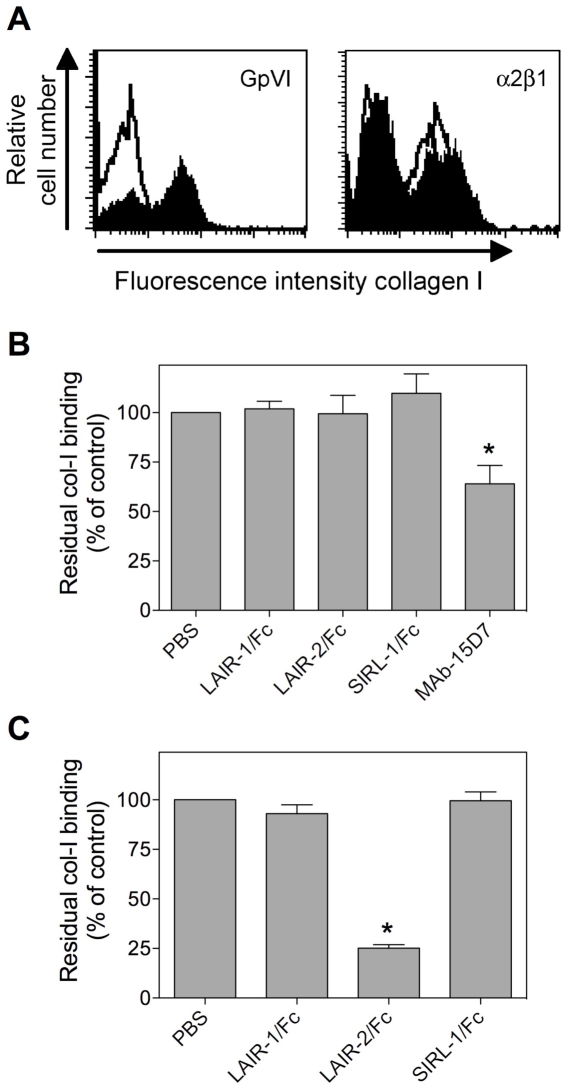
LAIR-2/Fc interferes with GpVI but not α2β1 binding to collagen. Jurkat-cells expressing GpVI or CHO-cells expressing α2β1 (Mn^2+^ activated) were incubated with FITC-labeled collagen I (7.7 µg/ml) in the absence or presence of 0.5 mg/ml LAIR-1/Fc, LAIR-2/Fc or SIRL-1/Fc. Residual collagen binding was subsequently analyzed via FACS-analysis. *Panel* A: representative histograms for binding of FITC-labeled collagen I to GpVI- or α2β1-expressing cells in the absence (closed histograms) or presence (open histograms) of LAIR-2/Fc. *Panel* B: Quantitative analysis of residual FITC-labeled collagen I binding to α2β1-expressing cells in the absence or presence of Fc-fusion proteins or the blocking α2β1 antibody MAb-15D7. *Panel* C: Quantitative analysis of residual FITC-labeled collagen I binding to GpVI-expressing cells in the absence or presence of Fc-fusion proteins. Data represent the relative mean fluorescent intensity (expressed as % of PBS control) ± SD of three independent experiments. *p<0.005 compared to control.

Various receptors support the adhesion of platelets to collagen surfaces, including GpVI, α2β1 as well as the VWF/GpIb axis. It was of interest therefore to investigate which of these pathways are inhibited by LAIR-2/Fc. First, cells expressing GpVI or α2β1 were incubated with FITC-labeled collagen (7.7 µg/ml) in the absence or presence of LAIR-1/Fc, LAIR-2/Fc or control fusion protein SIRL-1/Fc (0.5 mg/ml). Incubation with FITC-labeled collagen in the absence of these proteins resulted in significant binding to GpVI- or α2β1-expressing cells, as analyzed by flow cytometry (Fig. 4A). Binding of collagen to Mn^2+^-stimulated α2β1-expressing CHO-cells was unaffected by each of the fusion proteins tested, but was markedly reduced in the presence of the α2β1-blocking antibody 15D7 (Fig. 4B). Binding of collagen to GpVI-expressing cells was also unaffected by the presence of LAIR-1/Fc and SIRL-1/Fc. However, collagen binding was inhibited in the presence of LAIR-2/Fc (Fig. 4C).

Binding of VWF to collagen was addressed in an immunosorbent assay. Wells were coated with collagen type I or III (5 µg/well) and incubated with purified plasma-derived VWF (0.1 µg/ml) in the absence or presence of various concentrations fusion protein. Binding VWF to collagen I or III was similar in the absence or presence of LAIR-1/Fc or SIRL-1/Fc. In contrast, a dose-dependent inhibition of VWF to both types of collagen was observed in the presence of LAIR-2/Fc. Half-maximal inhibition was observed at 3 and 22 µg/ml LAIR-2/Fc for collagen type I and III, respectively. Thus, LAIR-2/Fc has the capacity to interfere with platelet-collagen interactions mediated by GpVI and VWF/GpIb.

## Discussion

Undesired exposure of vessel-wall components following disruption of the atherosclerotic plaque is often a prelude to coronary arterial thrombotic complications. Interactions between platelet collagen-receptors like GpVI, α2β1 or the GpIb/VWF complex and subendothelial collagen structures are central to the formation of platelet-rich thrombi that occlude the vasculature. In the present manuscript, we describe the use of a soluble human collagen-binding protein, *i.e.* LAIR-2 as a tool to interfere with platelet-collagen interactions. LAIR-2 nor its homologue LAIR-1 could be detected in platelets, either by FACS-analysis or Western blotting of whole platelet-lysates (Fig. 1). This suggests that in contrast to GpVI, α2β1 and VWF, both collagen-binding proteins are not expressed to a significant extent in platelets. This may seem surprising from the notion that LAIR-1 and LAIR-2 are expressed in numerous cells of hematopoietic origin, including CD34+ stem cells [17], [18]. Moreover, we recently established that LAIR-1 is surface-expressed in megakaryoblasts (T.A.M. Steevels, G.H.A. Westerlaken, M.R. Tijssen, P.J. Coffer, P.J. Lenting, J.W.N. Akkerman & L. Meyaard, manuscript under revision). LAIR-1 contains two ITIM-motifs, and its absence from platelets is possibly necessary to avoid the influx of conflicting (*i.e.* inhibitory *versus* activating) collagen-induced signals via LAIR-1 and the GpVI/FcRγ complex, respectively. Indeed, co-expression of LAIR-1 and GpVI results in silencing of collagen-induced signaling via GpVI [19].

**Figure 5:**
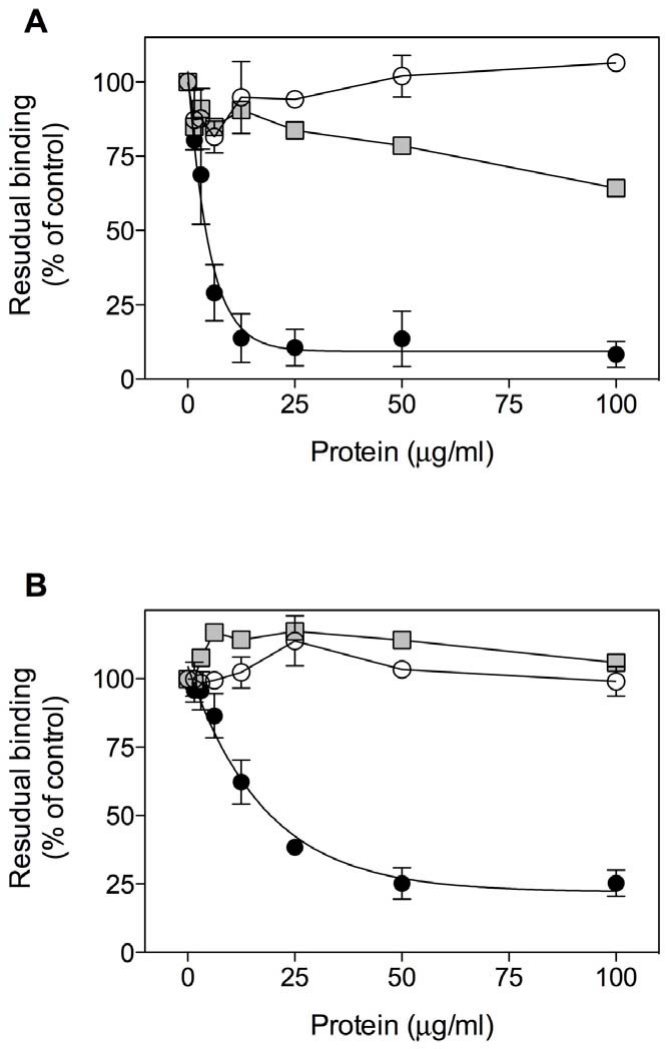
LAIR-2/Fc interferes with VWF binding to collagen. Microtiter wells were coated with 50 µg/ml collagen I (*panel* A) or collagen III (*panel* B) and subsequently incubated with 0.1 µg/ml purified plasma-derived VWF in the presence of increasing concentrations of LAIR-1/Fc (open circles), LAIR-2/Fc (closed circles) or SIRL-1/Fc (squares). VWF binding was detected with horseradish-conjugated polyclonal anti-human VWF antibodies and 3-3′-5-5′-tetramethylbenzidine. Data are representative of 3 independent experiments.

When added to PRP, LAIR-2/Fc but not LAIR-1/Fc was able to interfere with collagen-induced platelet aggregation (Fig. 2). LAIR-2/Fc mediated inhibition was found to be dose-dependent and specific, given that no inhibition was observed upon TRAP-induced platelet aggregation (Fig. 2). A LAIR-2/Fc specific inhibition of platelet-collagen interactions was also observed in *in vitro* perfusion experiments. The addition of LAIR-2/Fc to anticoagulated whole blood resulted in a dramatic decrease in the deposition of platelets when perfused over a collagen surface, both at low (300 s^−1^) and high (1500 s^−1^) shear rates (Fig. 3). Since different receptors dominate the interactions between platelets and collagen at low and high shear rates, our findings indicate that LAIR-2/Fc is able to interfere with the action of more than one collagen-receptor. Indeed, whereas LAIR-2/Fc was unable to interfere with the interaction between collagen and α2β1, LAIR-2/Fc but not LAIR-1/Fc inhibited binding of collagen to GpVI-expressing cells as well as binding of VWF to collagen (Figs. 4 and 5). These data are in agreement with the perfusion data, in that GpVI is important in the adhesion of platelets to collagen under low shear rate conditions, whereas VWF is pertinent to the adhesion of platelets to collagen under high shear rate conditions.

One unexpected observation in our study is the difference between LAIR-1/Fc and LAIR-2/Fc in their ability to interfere with collagen-platelet interactions. First, the primary structure of both proteins is highly homologous (>80% amino acid identity between LAIR-2 and the collagen-binding domain of LAIR-1) [20]. Second, both proteins display efficient binding to collagens [7], [9] Third, the collagen sequences recognized by LAIR-1 and LAIR-2 (as determined using the collagen tool-kits) overlap to a significant extent and collagen-binding is in both cases more efficient when the sequences are enriched in Glycine-Proline-Hydroxyproline (GPO)-triplets [10]. However, one important difference which was revealed by determining the collagen sequences that are being recognized by LAIR-1 and LAIR-2, is that LAIR-2 not only recognizes similar sequences as LAIR-1, but also a set of additional collagen-related motifs. This may explain why LAIR-2 and LAIR-1 behave differently with regard to platelet-collagen interactions.

The collagen toolkits have also been used to identify collagen sequences that are being recognized by α2β1, GpVI and VWF [21], [22], [23]. Both α2β1 and VWF recognize a single specific collagen sequence (GFOGER/N and RGQOGVMGF, respectively, with O being hydroxyproline), whereas GpVI seems to interact efficiently with sequences that contain multiple GPO-triplets. Since a similar preference for GPO-containing sequences has also been found for LAIR-2, this may provide a rationale for the inhibitory effect of LAIR-2/Fc on GpVI-collagen interactions. However, this does not explain why LAIR-2/Fc is able to interfere with VWF-collagen interactions, since the sequence recognized by VWF is not recognized by LAIR-2. Approximately 10 LAIR-2/Fc molecules bind per collagen monomer, whereas this amount is limited to one for VWF. The possibility exists that the high number of LAIR-2/Fc molecules (with a molecular weight of 82.5 kDa) prevent VWF from binding to collagen via steric hindrance.

In view of the efficient inhibition of collagen-dependent platelet adhesion and aggregation, it is of interest to speculate on the (patho)physiological consequences of our findings. Considering the low molecular weight of the genuine LAIR-2 protein (15–18 kDa) the protein is likely to have a very short half-life in the circulation. Indeed, using a sensitive antigen assay, LAIR-2 could not be detected in plasma of healthy individuals [9]. Thus, it seems inconceivable that LAIR-2 plays a relevant role in the regulation of platelet-collagen conditions under normal haemostatic conditions. However, LAIR-2 is mainly secreted by CD4^+^ T cells, suggesting that levels of LAIR-2 are locally increased at sites of inflammation. This is illustrated by increased levels of LAIR-2 in synovial fluid of patients with rheumatoid arthritis [24]. Interestingly, it has recently been reported that platelet-derived microparticles contribute to the pathogenesis of inflammatory arthritis in a GpVI-dependent manner [25]. It is tempting to speculate that LAIR-2 secreted by locally active CD4^+^ T cells could modulate GpVI-mediated disease progression.

Given the efficient manner in which LAIR-2 interferes with platelet-collagen interactions, also its therapeutical application as inhibitor of collagen-dependent thrombosis should be considered. In contrast to some of the collagen-inhibitors that are currently being developed, such as Saratin and Aegyptin, the risk of the formation of allo-antibodies is probably low in view of the human origin of the LAIR-2 protein.
